# Support Vector Machine Based Lane-Changing Behavior Recognition and Lateral Trajectory Prediction

**DOI:** 10.1155/2022/3632333

**Published:** 2022-05-10

**Authors:** Yingying Feng, Xiaolong Yan

**Affiliations:** College of Information Engineering, Fuyang Normal University, Fuyang 236041, China

## Abstract

With the development of technology, vehicle trajectory prediction and safety decision technology has become an important part of active safety technology. Among them, the vehicle trajectory prediction technology can predict the vehicle position, speed, and other motion states in the predicted period according to the current and historical vehicle running state, and the prediction results can provide support for judging the vehicle safety in the predicted period. In order to analyze the above problems, this study fully extracted the main feature information from the vehicle lane change track data with the help of the powerful nonlinear learning and high pattern recognition ability of support vector machine, and conducted identification modeling for the actual lane change process of the vehicle and predictive analysis of the vehicle lateral movement track. First, the lane-changing behavior of vehicles was analyzed, and the vehicle lane-changing execution stage and 10 influencing factors that could characterize lane-changing behavior were determined based on NGSIM to extract the data of lane-change-related variables. Then, a lane-changing recognition model based on gridsearch-PSO is proposed. In the Matlab environment, the model has a test accuracy of 97.68%, while the SVM model without optimization parameters has a recognition accuracy of only 80.87%. The results show that the model has strong classification ability and robustness. Finally, by using the polynomial model for lateral movement trajectory fitting, K-fold cross-validation method is used for fitting polynomial model fitting test.

## 1. Introduction

The driving safety of vehicles is of vital significance to drivers and pedestrians who participate in road traffic. With the increasing number of motor vehicles and drivers in China, the road driving environment becomes more complicated [1]. According to statistics, by the end of 2019, the number of cars in China has reached 220 million, and the number of drivers is about 397 million. The increasing number of motor vehicles and the uneven level of safety awareness of drivers make China's road environment more crowded and complex [2–4]. In 2018 alone, there were as many as 160,000 traffic safety accidents involving vehicles, with about 40,000 deaths and direct economic losses of 1.1 billion yuan [5, 6].

A large number of studies have shown that lane-changing behavior of drivers will affect the surrounding vehicles and thus increase the occurrence of accidents. Experts in the field of intelligent transportation in Germany have called for more research into lane-changing safety in order to reduce traffic safety problems caused by lane changing [7]. The vast majority of studies mainly focus on how to unlock the turning lane change behavior, but in the actual process, due to the low-signal stimulation rate, there is delay, mainly manifested as the lane change after the driver turns on the turn signal [8–11]. In addition, when the driver turns on the turn signal, it can only indicate his intention to change lanes or misoperation. In reality, the driver may not have started lane change or has already started lane change, which depends on the driving style of the driver and the actual road conditions [12]. Therefore, turning on the turn signal indicates that the driver starts to perform lane-changing operation which is not accurate and rigorous, while lane-changing behavior recognition based on the Internet of vehicles needs to determine the driver's lane-changing behavior in a short time, so higher requirements are put forward for the characterization of lane-changing behavior.

However, advanced driver assistance system is not always in the active state, ignoring the driver's own driving intentions [13, 14]. Therefore, when the intention of the vehicle driver is uncertain or contrary to its operating intention, the advanced driver assistance system may issue an alarm or autonomously control the vehicle on the one hand. Forced operation may reduce the driver's trust in the driver assistance system and bring greater psychological burden to the driver. On the other hand, reckless interference with moving vehicles tends to make drivers panic and eventually cause unnecessary traffic accidents. Therefore, it is necessary to consider the driver's intention and accurately identify the real-time intention of the driver. Lane change is a common driving behavior. This study studies the driver's lane-change intention and predicts the vehicle's lane-change trajectory on this basis, which provides theoretical support for the development and improvement of advanced driver-assistance system [15]. This section mainly focuses on the study of conventional vehicles, and the next section mainly introduces the lane-change problem of intelligent vehicles.

For intelligent vehicles, safety is the first priority. In the early design of vehicles, due to the limitation of sensing technology, vehicles could not carry out active safety protection, so passive safety systems such as airbags were mostly used to reduce the casualties in accidents [16, 17]. Though sensing technology has been significantly improved and the existing vehicle driving-assistant system is popular, it still can only make decisions according to the current traffic conditions, although can detect some dangerous on the road, make traffic safer, but on the road in the future there will not be able to effectively identify the potential dangers [18–20]. In order to detect the danger on the road in advance, it is necessary to have a certain cognitive ability to the future changes of the road environment around the vehicle, that is, to have a strong ability to predict the surrounding environment. The prediction of the surrounding environment by the intelligent driving system has always been a difficulty in the industry, especially in the intelligence of the bicycle. Because the field of vision of the sensor is blocked by the surrounding vehicles and other things, its detection range is limited, so the vehicle can get less environmental information, and the difficulty of prediction is further increased [21].

**Figure 1:**
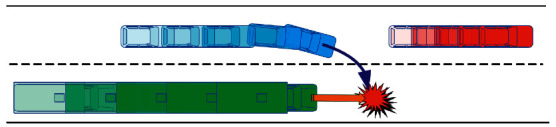
Schematic diagram of lane change risk of adjacent vehicles.

Vehicle trajectory prediction is an important part of environmental situation change. For manned vehicles, affected by the environment and the subjective thoughts, its future trajectory has certain uncertainty, unable to accurately predict the car position change vehicles in the lane changing easily occurs when the vehicle collision, so you need to predict in advance around vehicle lane-changing behavior, and then predict the lane changing trajectory. Determine in advance whether the lane change has adverse impact on the safety of the car [22]. As shown in Figure 1, when the car is inserted in front of the truck, the truck can only use emergency braking to avoid or minimize the harm, and the risk of collision between car and truck is very high. If the truck can predict that the neighboring vehicle is about to change lanes and insert into the front of the vehicle, the vehicle can smoothly slowdown in advance to avoid collision and ensure the driving comfort of the occupants. Therefore, the prediction of the vehicle's lane-change trajectory can improve the safety of driving.

The contribution of this paper is as follows:

1. At the same time, the theory and application of vehicle lane changing are studied
2. A lane-changing recognition model based on gridsearch-PSO is given for better accuracy
3. The k-fold cross validation method is used to fit the polynomial parameters so as to avoid the overfitting phenomenon

## 2. Related Work

Wu et al. [23] took into account the influence of increasingly complex urban environmental information on drivers' lane-changing decisions. Based on the data generated by driving simulators, they extracted two decision rules in the intention generation and implementation stages through rough set theory, providing a theoretical basis for lane-changing decisions in complex driving environments. Maguire et al. [24] combined hidden Markov model (HMM) and support vector machine (SVM) and collected parameters such as steering wheel angle accelerator pedal as the identification standard. Meanwhile, the results showed that the identification accuracy of this model was higher. In order to further improve the lane-change recognition rate, Shangguan et al. [25] built the RBF lane-change recognition model through normalized principal component analysis and genetic algorithm to continuously tune the parameters of the neural network, and further improved the recognition accuracy of the model by optimizing the model. Zhu et al. [26] recognized driving intentions by establishing LSTM method and calculated the probability of lane changing and going straight. Combined with NGSIM (Next Generation Simulation) data set, the model was trained and verified, and the test showed that the model had a significant effect on lane-changing intention identification.

Ma et al. [27] established the MGHMM model for driver lane-changing intention recognition, determined the state number and Gaussian mixture number of the model through experimental analysis, and analyzed the relationship between the time point of model recognition of lane-changing intention and observation data interception method and observation data category selection under single and compound working conditions. Based on SVM classification algorithm and based on steering angle, accelerator brake pedal, vehicle speed, acceleration and driver's line-of-sight information, Gao et al. [28] carried out research on driver's lane-changing intention identification and realized the prediction of driver's lane-changing intention 0.6s in advance, with a recognition accuracy up to 89%. Choi et al. [29] conducted overtaking experiments at 30, 40, 50, 60 km/h on urban roads and collected human face videos, vehicle data, and environmental information. The results show that the combination of feature variables (face/eye information + steering wheel angle entropy + vehicle state + environment information) can be used as the group of feature variables to characterize driving intention, and the hidden Markov model trained by the combination of feature variables can be used as the model to predict drivers' lane-change intention. Xie et al. [30] used polynomial to fit the lane-changing trajectory in the actual state of vehicles. Due to the difficulty in characterizing the beginning of lane-changing behavior, trajectory fitting results were better when the vehicle speed of lane changing was 20–30 m/s.

Lane track model focuses on lane-change process itself, aiming to predict vehicle lane-change trajectory or provide path planning for vehicle lane change. Lane-change trajectory model research needs a large amount of vehicle track data, making it start later than lane-change model. Benterki et al. [31] established the straight track function of surrounding vehicles by using the quintic polynomial and predicted the collision time between the surrounding vehicles and the main vehicle on the straight and curve, respectively, by using the parameter track curve. On the basis of the quintic polynomial trajectory planning, Zhang et al. [32] proposed the path planning of the quintic polynomial, which can realize the automatic parking function by using the fuzzy logic algorithm to track. The study also compared the path of the quintic polynomial with the B-spline curve and came to the conclusion that the former has better real-time performance and flexibility. In addition, the seventh-degree polynomial also increases the continuity of the curvature change rate of the trajectory curve, making vehicle trajectory tracking more comfortable. The polynomial lane-changing trajectory is characterized by continuous and differentiable curvature, so it is still an important method for scholars at home and abroad to study lane-changing trajectory. Xu et al. [33] used Bayesian network to model the driving behavior of vehicles, obtained the probability distribution of vehicle behavior, and made trajectory prediction by taking the uncertainty and random factors of behavior into account. The joint distribution of input and output is used to obtain the conditional probability density function, which can be used for trajectory prediction.

When the timing of lane change is not appropriate or the lane-change process itself and the surrounding operating environment changes, there may be a risk of traffic accidents if the lane change continues. Therefore, it is very important to study the correct lane-change safety decision for traffic safety. Reasonable lane-change safety decision will reduce the risk in the process of lane change, avoid the occurrence of dangerous lane-change behavior, give early warning to the possible collision accident, and make timely response to the changing lane-change environment to adjust the operation state of vehicles in the process of lane change. Zhang et al. [34] analyzed the generation of lane-changing motive and divided the lane-changing process into three stages according to the driver's steering wheel operation. Yuan et al. [35] divided lane-changing behavior into free lane-changing mandatory lane-changing cooperative lane-changing behavior, considered three lane-changing conditions of different types, and established vehicle dynamics models based on different lane-changing conditions in two-lane lane-changing scenarios using cellular automata to simulate and analyze the model. Chen et al. [36] divided lane-changing behavior into lane-changing motive to generate lane choice and to judge whether clearance is acceptable, and established a lane-changing choice model based on probability model.

## 3. Lane-Changing Behavior Recognition and Lateral Trajectory Prediction by Support Vector Machine

### 3.1. The Flow Chart of Vehicle Lane-Change Recognition

**Figure 2:**
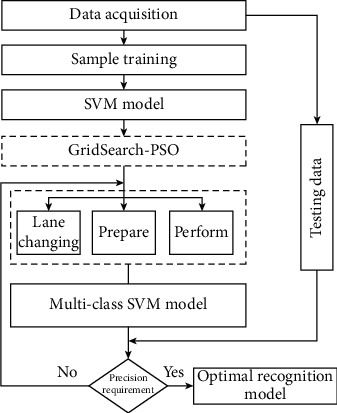
Flowchart of vehicle lane-change recognition in this study.

In support vector clustering and classification research, when constructing support function or category interface, it is expected to establish learning model with strong generalization ability through small sample learning to achieve the balance between structural risk and empirical risk. In recent years, it has been widely used in linear regression, pattern classification, prediction, and other fields. At present, lane changing, overtaking, and parallel intention recognition are used in traffic field. The whole system of the method is given in Figure 2.

The idea of vehicle lane-change recognition method is as follows: Firstly, the microvehicle driving track data is obtained, and the data is classified and preprocessed, and the parameters representing the characteristics of vehicle lane change are extracted as the sample data set. Secondly, SVM model was used to calibrate penalty parameter C and kernel parameter *G* using GridSearch-PSO to determine the optimal combination value.

**Step 1 .**

Data sample set acquisition. Through the microscopic driving data of each vehicle in the US-101 database of NGSIM, the lane-changing vehicles are screened out. From the perspective of driving track and combined with the influence of surrounding vehicles, the speed of the vehicle's position information along the driving direction is selected to be perpendicular to the driving direction, and the speed distance relative to the speed of the vehicle in front is selected.

**Step 2 .**

SVM model selection. Firstly, considering that the lane-changing process of the vehicle in this study is divided into three stages and the recognition results involved are of three categories, multiclass SVM model is selected.

**Step 3 .**

Parameter search and model training. The optimal combination of parameters is sought by optimizing the penalty parameter C and kernel parameter *G* in the SVM model with the grid Search-PSO algorithm.

**Step 4 .**

Model verification. After the training samples in step 3, the vehicle lane-change recognition model based on GridSearch-PSO optimization is established, and the test set samples are used to test the model.

### 3.2. SVM Model

SVM algorithm is to establish a classification hyperplane (decision surface) to solve the classification problem, so that the distance between the samples in the training data set and the classification hyperplane can reach the maximum. For any sample, satisfy

(1) $$\begin{array}{c} \left\{\begin{array}{cc} w^{T}x_{i}+b\geq +1, & y_{i}=+1,\\ w^{T}x_{i}+b\leq -1, & y_{i}=-1. \end{array}\right. \end{array}$$

Maximizing *D* to meet the equation below

(2) $$\begin{array}{c} \underset{w,b}{\max}\frac{2}{\left\|w\right\|},\\ y_{i}\left(w\cdot x_{i}+b\right)\geq 1,\;i=1,2,\ldots ,l. \end{array}$$

In order to obtain the optimization objective, the maximization problem of equation (2) is equivalent to the minimization problem, i.e.,

(3) $$\begin{array}{c} \underset{w,b}{\max}\frac{1}{2}{\left\|w\right\|}^{2},\\ y_{i}\left(w\cdot x_{i}+b\right)\geq 1,\;i=1,2,\ldots ,l. \end{array}$$

By constructing Lagrangian multiplier solution formula (3), the Lagrangian function of this problem is obtained:

(4) $$\begin{array}{c} L\left(w,b,a\right)=\frac{1}{2}{\left\|w\right\|}^{2}+\sum_{i=1}^{l}\alpha_{i}\left[1-y_{i}\left(w^{T}x_{i}+b\right)\right]. \end{array}$$

The duality problem of equation (3) can be obtained by combining the above equation:

(5) $$\begin{array}{c} \left\{\begin{array}{c} \underset{\alpha }{\max}\overset{l}{\underset{i}{\sum }}\alpha_{i}-\frac{1}{2}\overset{l}{\underset{i=1}{\sum }}\overset{l}{\underset{j=1}{\sum }}\alpha_{i}\alpha_{j}y_{i}y_{j}x_{i}^{T}x_{j},\\ \text{s.t. }\overset{l}{\underset{i=1}{\sum }}\alpha_{i}y_{i}=0,\\ \alpha_{i}\geq 0,\;i=1,2,\ldots ,l. \end{array}\right. \end{array}$$

And the solution is

(6) $$\begin{array}{c} f\left(x\right)=w^{T}x+b=\sum_{i=1}^{l}\alpha_{i}y_{i}x_{i}^{T}x+b. \end{array}$$

Based on (6), the lane-change preparation and lane-change execution in multiclass SVM are given (7).

### 3.3. Multiclass SVM

The support vector machines introduced above are all standard support vector machines, which can only solve the binary classification problem. However, the behavior state in the process of vehicle lane changing is a multiclassification problem. SVM essentially solves the binary classification problem and cannot be directly used for vehicle lane-changing classification and recognition. Therefore, it is necessary to construct a multiclassification machine, the lane-changing behavior of vehicles was identified by the method of class against class, and the behavior states of vehicles were divided into three categories: lane-change following, lane-change preparation, and lane-change execution:

(7) $$\begin{array}{c} \left\{\begin{array}{c} \underset{w^{ij},b^{j},\xi^{j}}{\min}\frac{1}{2}w^{ij}\cdot w^{ij}+C\overset{3}{\underset{t=1}{\sum }}\xi_{t}^{ij},\\ \text{s.t.}\left\{\begin{array}{cc} w^{ij}\cdot \varphi \left(x_{t}\right)+b^{ij}\geq 1-\xi_{t}^{ij}, & \text{if }y_{t}=i\\ w^{ij}\cdot \varphi \left(x_{t}\right)+b^{ij}\leq -1+\xi_{t}^{ij}, & \text{if }y_{t}=j\\ \xi_{t}^{ij}\geq 0, & i,j=1,2,3 \end{array}.\right. \end{array}\right. \end{array}$$

The classifier decision function constructed from the data of category *I* and *j* is

(8) $$\begin{array}{c} f_{ij}\left(x\right)={\left(w^{i,j}\right)}^{T}\phi \left(x\right)+b^{i,j}. \end{array}$$

The solution can be obtained by combining the above formula:

(9) $$\begin{array}{c} f\left(x\right)=w^{T}\phi \left(x\right)+b\\ =\sum_{i=1}^{l}\alpha_{i}y_{i}\phi {\left(x_{i}\right)}^{T}\phi \left(x\right)+b\\ =\sum_{i=1}^{l}\alpha_{i}y_{i}K\left(x_{i},x\right)+b. \end{array}$$

Commonly used kernel functions mainly have the following types:

(10) $$\begin{array}{c} K\left(x_{i},x_{j}\right)=x_{i}^{T}x_{j}. \end{array}$$

The weight matrix is defined as follows:

(11) $$\begin{array}{c} K\left(x_{i},x_{j}\right)={\left(x_{i}^{T}x_{j}+d\right)}^{q},\\ K\left(x_{i},x_{j}\right)=\exp\left(-\frac{{\left\|x_{i}-x_{j}\right\|}^{2}}{2\sigma^{2}}\right). \end{array}$$

**Figure 3:**
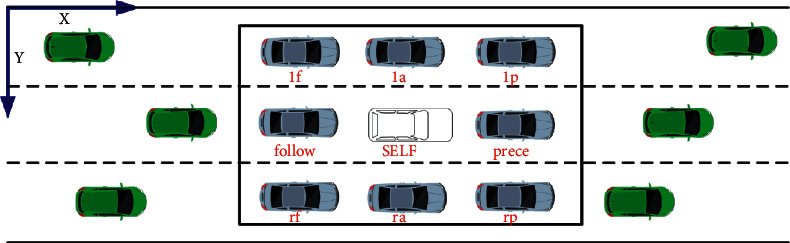
The schematic diagram of adjacent vehicles.

From the above analysis, the multiclass SVM proposed is used to solve the adjacent vehicles shown in Figure 3:

## 4. Experimental Results and Analysis

### 4.1. Introduction to Experimental Data Set

The original NGSIM data can be directly used in model experiments after data cleaning. The data used in this study is the track data of cleaned vehicles, with a total of 2056 sample data, including 1028 lane changing samples and 1028 samples that keep straight driving with a lane-changing time of 10 s and a sampling interval of 0.1 s. The total sample data size is 205600. In this study, 80% of the samples were used as training data, 10% as validation data, and the remaining 10% as test data. Therefore, the total training data is 164,480 pieces, and the verification data and test data are 20,560 pieces.

The data used in this study are the SECTIONS of US-101 expressway in NGSIM data set. The section acquisition area is shown in Figure 3, with a total length of about 2100 feet (640 meters). The section containing five main carriageways; one auxiliary lane and an incoming and outgoing ramp with a width of 12 feet (3.66 meters) was successively equipped with eight cameras to collect detailed track data of vehicles. During the data detection, the track data of the process of traffic volume passing through the section from unblocked to crowded was recorded in detail.

### 4.2. Experimental Results Analysis

The multiclass SVM training model was established by using the LibSVM tool box in Matlab environment. Before the training model, it is necessary to calibrate the parameter combination of RBF kernel function to seek the optimal combination value of kernel parameter *G* and penalty parameter *C* in the kernel function, so as to train SVM classification, and the model can recognize lane-changing status of vehicle lane-changing data. In this study, grid search and particle swarm optimization are used to optimize the parameters.

The optimal parameter combination is sought to ensure a certain search length, and the value range of the initial parameters *C* and *G* is set as [2–10, 2 10]. The grid search algorithm can decouple the parameters *C* and *G* in the kernel function with high parallelism, but the calculation time is too long because the parameter search range is too large. At the same time, the PSO algorithm is easy to fall into local optimization and the GridSearch algorithm takes a long time. When Best*C* = 4, *g* = 12.1257, the accuracy of the training model can reach 98.5%.

**Figure 4:**
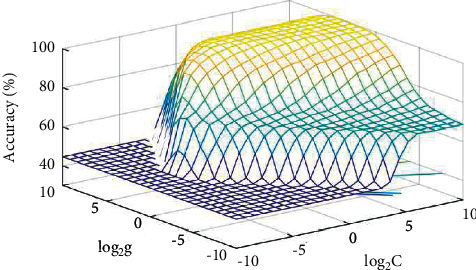
GridSearch-PSO parameter selection.

The parameter selection process using GridSearch-PSO method is shown in Figure 4. Accuracy represents the classification accuracy, and Best*C* and *G* represent the parameter combination under the optimal classification Accuracy.

**Figure 5:**
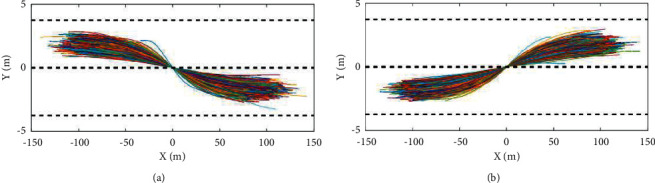
Change lanes to the left and right typical trajectory.

For lane-change behavior, due to the constraints of highway road structure, vehicles will not move randomly in the highway. On the contrary, lane-change trajectory always follows the established pattern, so it is possible to directly model the shielded lane-change trajectory form based on inverted time, and reserve enough time for the required actions such as testing and joint adjustment, thus ensuring the complete presentation of large-scale integrated game information system functions. Typical left-lane change tracks and right-lane change tracks obtained by screening are shown on the left and right sides of Figure 5.

So, we need to optimize and upgrade the system. In the real world, there is no very clear boundary between the beginning and end of the behavior of lane-changing subjects, which can be classified as the previous stage or the next stage depending on the situation. The selection of time duration is related to the quality of the screened lane-change trajectory. Therefore, the screened lane-change trajectory length (the length of the time series of lane-change trajectory) should be selected so that the screened lane-change trajectory can cover the basic typical lane-change trajectory under the condition of such time duration.

In most papers, the three lane-changing styles are called calm common style and radical style in order: calm common style and radical style. According to the number of samples assigned by clustering under each category, vehicles running on expressways tend to prefer normal and calm lane-changing styles. Subjectively, the safety of these two lane-changing styles can be analyzed. The aggressive driving lane-changing style will frequently change lanes to overtake, increasing the risk of driving.

**Figure 6:**
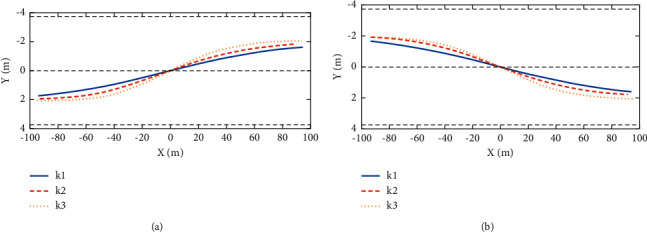
Left- and right-lane change prototype trajectory.

Under each lane-change style, there are many sample tracks, and there are small differences between the tracks of the same lane-change style. Therefore, in order to facilitate the modeling of lane-change trajectory, the prototype trajectory needs to be obtained. In this study, tracks of different lane-changing styles are averaged, and the data obtained are used as the basis for modeling. The prototype tracks of left- and right-lane changing are shown on the left and right sides of Figure 6. When the number of samples of left-lane change is 334 and the number of samples of right-lane change is 354, the proposed method has the best effect.

**Figure 7:**
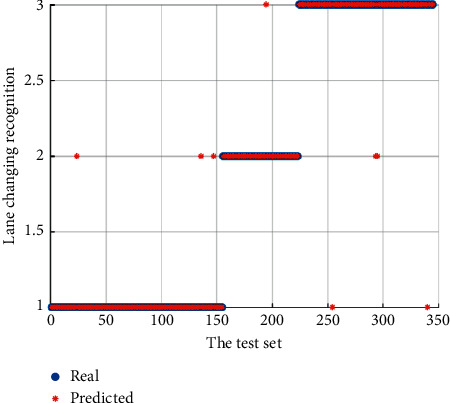
Sample test set lane-change recognition results.

In order to further verify the applicability of model parameters, the remaining 345 groups of data were taken as sample test set and classified and identified by the model. Lane-change recognition results of the sample test set are shown in Figure 7. Data points in the following stage of the vehicle have three classification errors, and data points in the lane-change preparation stage have one classification error. There are two classification errors in the data points during lane change, and the recognition accuracy can reach 98%.

**Figure 8:**
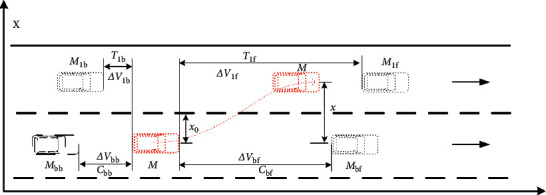
Microfactors affecting lane-change time of vehicles.

The micro-lane-changing trajectory behavior of vehicles is studied in this work. That is, it is assumed that vehicles are in a relatively normal driving environment in the macroenvironment, and macrofactors such as traffic flow density are not taken into account, and the microdriving characteristics of vehicles in the lane-changing process are taken into account, as shown in Figure 8. The distance difference and relative speed between the vehicle changing lane and the vehicle in front in the current lane are the potential factors affecting lane changing time.

## 5. Conclusion

Today, the world is vigorously developing intelligent and intelligent vehicle's understanding of the surrounding environment is the premise of decision-making and control. Trajectory prediction is an important part of it. Trajectory prediction of surrounding vehicles in advance can analyze future road risks and update path planning in real time. In this study, the behavior of adjacent traffic vehicles is identified, and the advantages of different models in short- and long-term prediction are combined with the interactive multimodel of traffic vehicles.

At the same time, the correlation between lane-change time and lane change lateral trajectory was analyzed, and the prediction model of lane change lateral trajectory was built. Finally, the prediction model is validated with test data samples.

Due to the great challenges of trajectory prediction and the limitations of research time and working conditions, there are still many problems to be solved and studied: (1) Highway scenario is a relatively simple road scenario, but the traffic environment in the real world is very complex. The method in this work does not consider pedestrians, complex scenarios, such as traffic jam, the algorithm of adaptive have a definite limit In order to enhance the adaptability of this method, need to further improve the (2) track prediction algorithm is proposed in this paper does not take into account the local and surrounding vehicles and environment related characteristics of volatility, but in the real world, the on-board sensors on the road information acquisition is fluctuating, this paper puts forward the track prediction algorithm should have certain robustness, and can when there is a deviation in the state of the information, track prediction effect is still able to meet the requirements, so need to predict algorithm robustness for related research (3) based on the trajectory prediction, the transverse longitudinal trajectory decoupling. Forecast on the longitudinal, handled by assuming that the vehicle speed constant, although this method has certain feasibility, but the reality vehicle lane changing, the speed is volatile, if can take into consideration of the uncertainty of vehicle longitudinal velocity, the forecast track will be more precise, to better serve the smart car enough policy makers.

## Data Availability

The data used to support the findings of this study are available from the corresponding author upon request.
